# DWI-related texture analysis for prostate cancer: differences in correlation with histological aggressiveness and data repeatability between peripheral and transition zones

**DOI:** 10.1186/s41747-021-00252-y

**Published:** 2022-01-12

**Authors:** Chie Tsuruta, Kenji Hirata, Kohsuke Kudo, Naoya Masumori, Masamitsu Hatakenaka

**Affiliations:** 1 Department of Diagnostic Radiology, School of Medicine, Sapporo Medical University, South 1, West 17, Chuo-ku, Sapporo, 060-8556 Japan; 2 Department of Diagnostic Imaging, Graduate School of Medicine, Hokkaido University, Sapporo, Japan; 3 Global Center for Biomedical Science and Engineering, Faculty of Medicine, Hokkaido University, Sapporo, Japan; 4 Department of Urology, School of Medicine, Sapporo Medical University, Sapporo, Japan

**Keywords:** Diffusion magnetic resonance imaging, Image interpretation (computer-assisted), Neoplasm grading, Prostate neoplasms, Reproducibility of results

## Abstract

**Background:**

We investigated the correlation between texture features extracted from apparent diffusion coefficient (ADC) maps or diffusion-weighted images (DWIs), and grade group (GG) in the prostate peripheral zone (PZ) and transition zone (TZ), and assessed reliability in repeated examinations.

**Methods:**

Patients underwent 3-T pelvic magnetic resonance imaging (MRI) before radical prostatectomy with repeated DWI using *b*-values of 0, 100, 1,000, and 1,500 s/mm^2^. Region of interest (ROI) for cancer was assigned to the first and second DWI acquisition separately. Texture features of ROIs were extracted from comma-separated values (CSV) data of ADC maps generated from several sets of two *b*-value combinations and DWIs, and correlation with GG, discrimination ability between GG of 1–2 *versus* 3–5, and data repeatability were evaluated in PZ and TZ.

**Results:**

Forty-four patients with 49 prostate cancers met the eligibility criteria. In PZ, ADC 10% and 25% based on ADC map of two *b*-value combinations of 100 and 1,500 s/mm^2^ and 10% based on ADC map with *b*-value of 0 and 1,500 s/mm^2^ showed significant correlation with GG, acceptable discrimination ability, and good repeatability. In TZ, higher-order texture feature of busyness extracted from ADC map of 100 and 1,500 s/mm^2^, and high gray-level run emphasis, short-run high gray-level emphasis, and high gray-level zone emphasis from DWI with *b*-value of 100 s/mm^2^ demonstrated significant correlation, excellent discrimination ability, but moderate repeatability.

**Conclusions:**

Some DWI-related features showed significant correlation with GG, acceptable to excellent discrimination ability, and moderate to good data repeatability in prostate cancer, and differed between PZ and TZ.

**Supplementary Information:**

The online version contains supplementary material available at 10.1186/s41747-021-00252-y.

## Key points

• Some diffusion-weighted imaging (DWI)-related texture features significantly correlated with histological aggressiveness in prostate cancer.

• Some DWI-related texture features show clinically acceptable data repeatability in prostate cancer.

• Texture features showing correlation with histological aggressiveness and repeatability differ between zones.

• DWI with *b*-values of 100 and 1,500 s/mm^2^ may be relevant.

## Background

Texture analysis of clinical imaging has been increasingly carried out to determine its correlation with histological findings, such as lesion aggressiveness and clinical outcome [1–3]. Texture features extracted from magnetic resonance diffusion-weighted imaging (DWI), including apparent diffusion coefficient (ADC) maps, have shown promising results. However, there is no consensus regarding the method to calculate DWI-related metrics such as monoexponential fitting, intravoxel incoherent motion, and diffusion kurtosis imaging. From a clinical perspective, ADC maps calculated from two different *b*-values can be simple and easy to use, but there is no consensus regarding the use of a combination of two *b*-values. Furthermore, there are concerns regarding the reliability of texture features which are sensitive to imaging characteristics, possibly having coincidental significance due to a larger number of parameters [4–6].

Magnetic resonance imaging (MRI) is a primary imaging modality used for prostate cancer. Many studies have been reported regarding the correlation between DWI-related parameters and lesion aggressiveness, such as the Gleason score (GS) and grade group (GG), with inconsistent results. It was reported that ADC entropy showed significant difference between GS of 3 + 4 and 4 + 3 but not in ADC mean [7]. Alessandrino F et al. [8] reported similar results, with no significance in ADC mean. In contrast, Itou Y et al. [9] reported that ADC median showed a significant correlation with GS and a significant difference between GS of 3+4 and 4+3. Shan Y et al. [10] reported that ADC mean showed a significant correlation with GS and a significant difference between GS of 3 + 4 and 4 + 3. Though some studies evaluated data reliability focusing on intraobserver and interobserver agreement for the same images (ADC maps) [11, 12], few studies have been performed with respect to image data reliability itself. Furthermore, though the above studies dealt with cancers in the peripheral zone (PZ) and transition zone (TZ) together, Hambrock T et al. [13] reported that ADC median showed a significant correlation with Gleason grade in PZ cancer. Jyoti R et al. [14] also reported that ADC minimum was significantly correlated with GS in PZ cancer, but not in TZ cancer. These results raise the possibility that DWI-related features may demonstrate a different relationship with tumor aggressiveness between the PZ and TZ. In a recent systematic review, Surov A et al. [15] reported that in PZ cancer, ADC moderately correlates with GS, but it weakly correlated with in TZ cancer.

This study aimed to analyze the correlation between texture features extracted from ADC maps generated from several sets of two *b*-value combinations or DWIs with several *b*-values, and GG in the PZ and TZ, separately, and to evaluate the reliability of texture features in repeated examinations.

## Methods

### Population, inclusion, and exclusion criteria

**Fig. 1:**
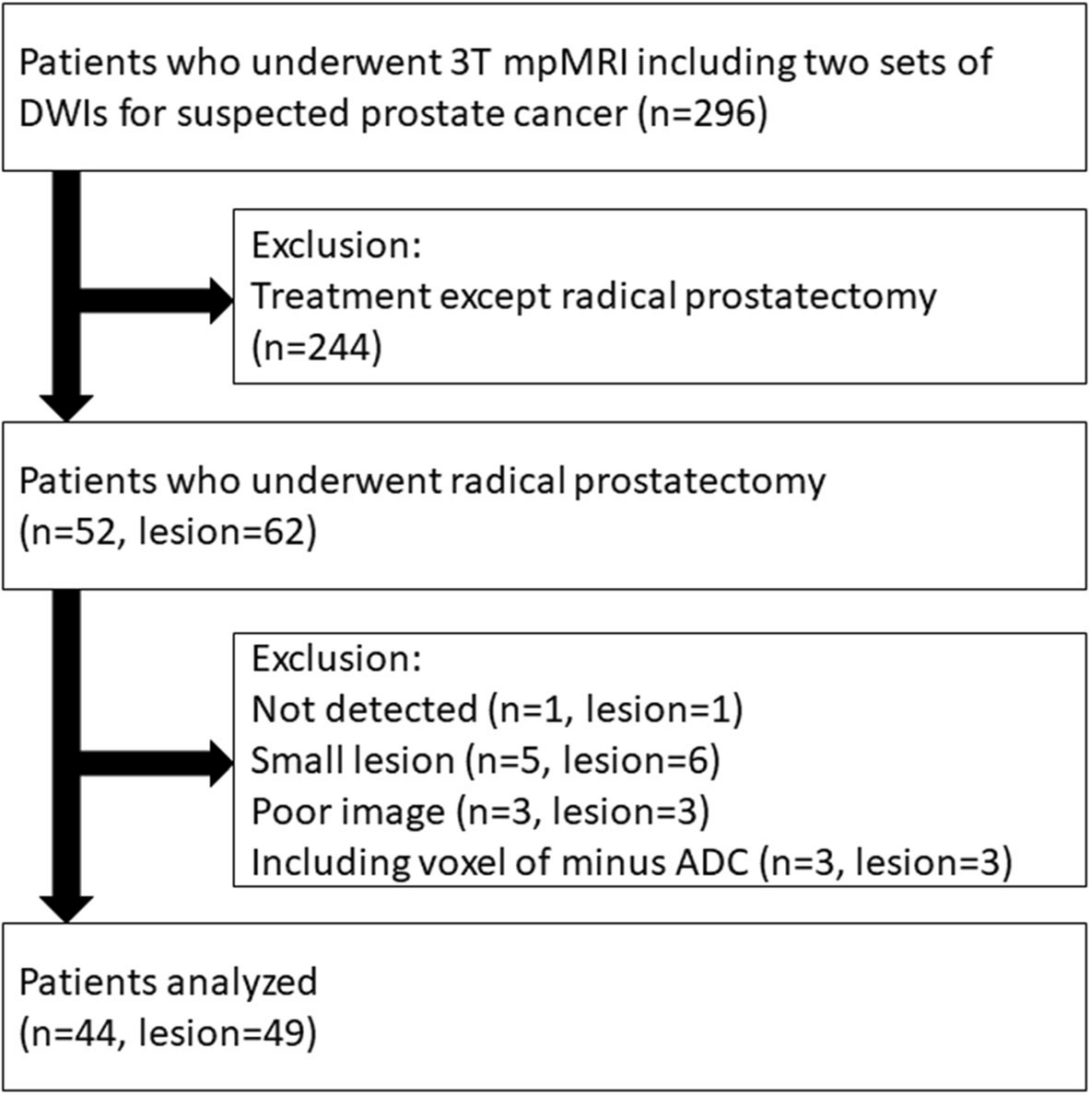
Flowchart of study showing inclusion and exclusion criteria, and patient and lesion numbers

This study was compliant with Helsinki Declaration. The following inclusion and exclusion criteria were considered: Inclusion criteria: patients who underwent 3-T multiparametric MRI (mpMRI) at our institute, including two sets of repeated DWI acquisitions for evaluating prostate lesions with informed consent from July 2016 to May 2020. Exclusion criteria: treatment except radical prostatectomy; lesions with a longitudinal diameter < 10 mm; lesions not detected on DWI; lesions with a voxel number within the region of interest (ROI) < 50; lesions containing voxel with ADC value < 0; poor image quality. Figure 1 shows the flowchart of patient inclusion and exclusion.

### MRI

MRI was performed using a 3-T system (Ingenia, Philips Healthcar, Eindhoven, The Netherlands) with a pelvic phased-array coil. No endorectal coil was used. Either 20-mg hyoscine-N-butyl-bromide or 1-mg glucagon was injected intramuscularly before examination to minimize bowel peristalsis.

A routine mpMRI protocol was applied to all patients, including sagittal, coronal, and axial T2-weighted imaging; axial DWIs; and axial dynamic contrast-enhanced imaging before and after gadolinium chelate injection of 0.1 mmol/kg gadoterate meglumine, Magnescope, Dotarem (Guerbet, Villepinte, France). For DWI, two sequential free-breathing DWI single-shot spin-echo echo-planar images were acquired. The patient remained in the same position between the two DWI acquisitions. Four *b*-values (0, 100, 1,000, and 1,500 s/mm^2^) with three orthogonal diffusion probing gradients were generated. ADC maps were generated using DWIs with *b*-values of 100 and 1,000 s/mm^2^, ADC map (100, 1,000) in line with the Prostate Imaging–Reporting and Data System (PI-RADS) version 2.1 (https://www.acr.org/-/media/ACR/Files/RADS/PI-RADS/PIRADS-V2-1.pdf) for the first and second DWIs, respectively. The DWI sequence parameters are summarized in Supplemental Table S1.

### Image analysis

**Fig. 2:**
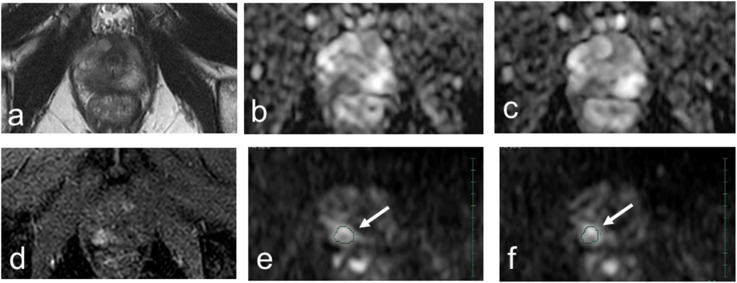
Multiparametric magnetic resonance imaging of the case (70 years, right peripheral zone cancer, GG of 3, PI-RADS of 4, T2aN0M0). **a** Axial T2-weighted image (repetition time of 4,000 ms and echo time of 80 ms). **b** First axial apparent diffusion coefficient (ADC) map (100, 1,000). **c** Second axial ADC map (100, 1,000). **d** Dynamic contrast-enhanced T1-weighted image. **e** First DWI 1,500. **f** Second DWI 1,500. Arrows indicate polygonal areas of the region of interests on **e** and **f**

**Fig. 3:**
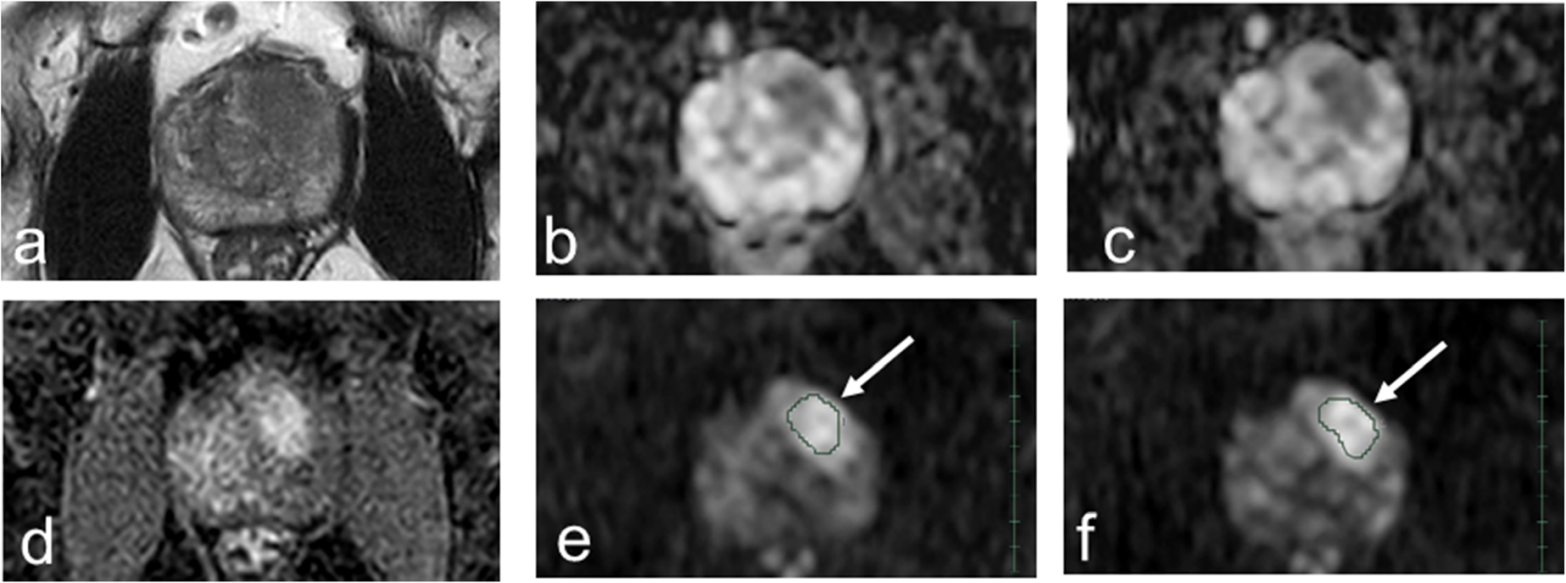
Multiparametric magnetic resonance imaging of the case (61 years, transition zone cancer, GG of 2, PI-RADS of 5, T2cN0M0). **a** Axial T2-weighted image (repetition time of 4,000 ms and echo time of 80 ms). **b** First axial apparent diffusion coefficient (ADC) map (100, 1,000). **c** Second axial ADC map (100, 1,000). **d** Dynamic contrast-enhanced T1-weighted image. **e** First DWI 1,500. **f** Second DWI 1,500. Arrows indicate polygonal areas of the region of interests on **e** and **f**

Image analysis including ROI assignment was performed by a consensus decision of two observers (C.T. and M.H. with 4 and over 30 years of experience in diagnostic radiology, respectively) using a Synapse Vincent 3D Image Analysis System (Fujifilm Corporation, Tokyo, Japan). For PZ cancer, the polygonal two-dimensional ROI was manually determined on the lesion in the center slice showing hyperintensity on the first DWI with a *b*-value of 1,500 s/mm^2^ (DWI 1,500) and hypointensity on the first ADC map (100, 1,000), referring to T2-weighted imaging, dynamic contrast-enhanced imaging, and whole-mount, step-sectioned histological evaluation of prostatectomy specimen. Then, the ROI was placed on the first DWI datasets of DWI 0, DWI 100, and DWI 1,000 through IVIM application of a Synapse Vincent 3D Image Analysis System. For non-peripheral transition zone (TZ) cancers, the polygonal two-dimensional ROI was manually determined on the lesion in the center slice showing hypointensity on T2-weighted images and hyperintensity on the first DWI 1,500, referring to the first ADC map (100, 1,000), dynamic contrast-enhanced imaging, and whole-mount, step-sectioned histological evaluation of prostatectomy specimen. After this, the ROI was placed on the first DWI datasets of DWI 0, DWI 100, and DWI 1,000 through intravoxel incoherent motion (IVIM) application of the Synapse Vincent 3D Image Analysis System. The same procedures were repeated for the second DWI datasets. Voxel data distributions within the ROI were rendered in comma-separated values (CSV) format (Supplemental Figs. S1 and S2) using a Synapse Vincent 3D Image Analysis System. Then, the ADC of each voxel was calculated by fitting signal intensity decay between four patterns of *b*-value combinations using a monoexponential curve fit: 0 and 1,000 s/mm^2^, ADC (0, 1,000); 0 and 1,500 s/mm^2^, ADC (0, 1,500); 100 and 1,000 s/mm^2^, ADC (100, 1,000); and 100 and 1,500 s/mm^2^, ADC (100, 1,500). Representative cases are shown in Figs. 2 and 3.

We assigned a two-dimensional ROI in the center slice of the lesion because more than half lesions were not large enough to place a three-dimensional ROI. Only 21 lesions, 43% of total lesions, showed longitudinal diameter > 12 mm on images and could be determined on equal to or more than four slices (DWI slice thickness of 3 mm/ gap of 0 mm, Supplemental Table 1) that would have satisfied assigning ROIs on two or more slices avoiding peripheral images, possibly being affected by partial volume effect. Texture analysis calculates the relationship between adjacent voxels, and thus, we assumed that appropriate texture analysis required at least four voxels along each direction.

All voxels within the ROI were extracted from the CSV data of ADC (0, 1,000), (0, 1,500), (100, 1,000), and (100, 1,500), and DWI 0, 100, 1,000, and 1,500. First-order statistical variables (minimum, 10%, 25%, median, 75%, 90%, maximum, mean, sum, standard deviation, skewness, kurtosis, energy, and entropy) were calculated. After discretization of voxel values (bin number 32), higher-order texture analysis was performed in a two-dimensional manner to generate a gray-level co-occurrence matrix (GLCM), gray-level run-length matrix (GLRLM), gray-level zone-size matrix (GLZSM), and neighborhood gray-level difference matrix (NGLDM). Homogeneity, energy, correlation, contrast, entropy, and dissimilarity were calculated from the GLCM. Short-run emphasis (SRE), long-run emphasis (LRE), low gray-level run emphasis (LGRE), high gray-level run emphasis (HGRE), short-run low gray-level emphasis (SRLGE), short-run high gray-level emphasis (SRHGE), long-run low gray-level emphasis (LRLGE), long-run high gray-level emphasis (LRHGE), gray-level non-uniformity for run (GLNUr), run-length non-uniformity (RLNU), and run percentage (RP) were calculated from GLRLM. Short-zone emphasis (SZE), long-zone emphasis (LZE), low gray-level zone emphasis (LGZE), high gray-level zone emphasis (HGZE), short-zone low gray-level emphasis (SZLGE), short-zone high gray-level emphasis (SZHGE), long-zone low gray-level emphasis (LZLGE), long-zone high gray-level emphasis (LZHGE), gray-level non-uniformity for zone (GLNUz), zone length non-uniformity (ZLNU), and zone percentage (ZP) were calculated from GLZSM. Coarseness, contrast, and busyness were calculated from NGLDM. Texture features were computed using the PTexture package (www.github.com/metavol/ptexture) written in Python language. The detailed methods are described elsewhere [16].

### Statistical analysis

Statistical analyses were carried out separately for PZ and TZ cancers. First, the correlation between texture features and GG was evaluated using Spearman's rank correlation test. For the features showing significance at both the first and second examinations, receiver operating characteristic (ROC) curves for differentiating between GG of 1 and 2 *versus* GG of 3, 4, and 5 were drawn, and the area under the curve (AUC) was calculated because there was a difference in prognosis between GG of 1 and 2, *versus* GG of 3, 4, and 5 [17]. To check test-retest data repeatability, intraclass correlation coefficient (ICC) and Bland-Altman plot (%) (% difference was used to normalize differences in original data magnitude) were used. Statistical analyses were performed using GraphPad Prism ver. 7.05 (GraphPad Software, San Diego, USA) and SPSS statistics ver. 25 (International Business Machines Corporation, Armonk, USA); *p*-values < 0.05 were considered statistically significant.

We considered the following values for classifying the strength of correlation: moderate (|ρ|: 0.4-0.7), strong (|ρ|: 0.7-0.9), and very strong (|ρ|: 0.9-1) [18], discrimination ability: acceptable (AUC: 0.7–0.8), excellent (AUC: 0.8–0.9), and outstanding (AUC > 0.9) [18], and data repeatability: moderate (ICC: 0.5–0.75), good: (ICC: 0.75–0.9), and excellent (ICC: 0.9–1) [19].

## Results

From July 2016 to May 2020, a total of 296 patients with suspected prostate cancer underwent mpMRI including two sets of repeated DWI acquisitions for evaluating prostate lesions with informed consent. Among them, 52 patients underwent mpMRI before prostatectomy and were histologically diagnosed as prostate cancer by the institutional pathologists. There were 62 cancers with a longitudinal diameter ≥ 10 mm. Furthermore, one lesion undetected on DWI, six lesions with a voxel number within the ROI < 50, three lesions with poor image quality either in the first or second DWI, and three lesions containing voxel with ADC value < 0 were excluded. Finally, 44 patients with 49 cancers were analyzed. The characteristics of patients and lesions are summarized in Table 1. Of them, 11 patients underwent prostate biopsy within 6 weeks (17−36 days) before mpMRI, but no clear adverse effects were included for analysis. The duration between MRI and radical prostatectomy was from 8 to 191 days (median 69 days).

**Table 1:** Summary of patient and lesion characteristics

Parameter	Value
No. of patients	44
Median age (year), (range)	68 (49–79)
No. of cancers	49
PI-RADS 2.1 score	
3	1
4	38
5	10
Grade group	
1	1
2	27
3	16
4	3
5	2
Location of cancer	
Peripheral zone	30
Not peripheral zone (Transition zone)	19
Number of voxels included in the ROI	
First, median, (range)	104 (58–231)
Second, median, (range)	101 (51–271)

*PI-RADS* Prostate Imaging–Reporting and Data System, *ROI* Region of interest

As a representative of ADC histograms, ADC 10% calculated from ADC (0, 1,000), (0, 1,500), (100, 1,000), and (100, 1,500) are summarized in Table 2 and classified according to the PZ and TZ as well as GG.

**Table 2:** Summary of the breakdown of ADC 10% according to grade group and the *b*-value combination in peripheral and transition zones

Zone/grade group		Combination of two *b*-values × s/mm^2^			
		(0, 1,000)	(0, 1,500)	(100, 1,000)	(100, 1,500)
Peripheral zone		First/second of ADC 10% × 10^-3^ mm^2^/s			
Grade group	1	0.763/0.869	0.663/0.743	0.760/0.857	0.648/0.694
	2	0.767/0.782	0.660/0.678	0.651/0.694	0.584/0.612
	3	0.595/0.633	0.525/0.572	0.505/0.541	0.452/0.490
	4	0.609/0.654	0.537/0.599	0.557/0.558	0.482/0.532
	5	0.529/0.517	0.431/0.451	0.454/0.438	0.386/0.397
Transition zone		First/second of ADC 10% ×10^-3^ mm^2^/s			
Grade group	1	–	–	–	–
	2	0.708/0.676	0.626/0.598	0.636/0.584	0.566/0.536
	3	0.745/0.744	0.659/0.658	0.700/0.637	0.610/0.555
	4	0.748/0.733	0.682/0.653	0.645/0.728	0.656/0.573
	5	0.741/0.773	0.636/0.634	0.638/0.592	0.456/0.563

**Fig. 4:**
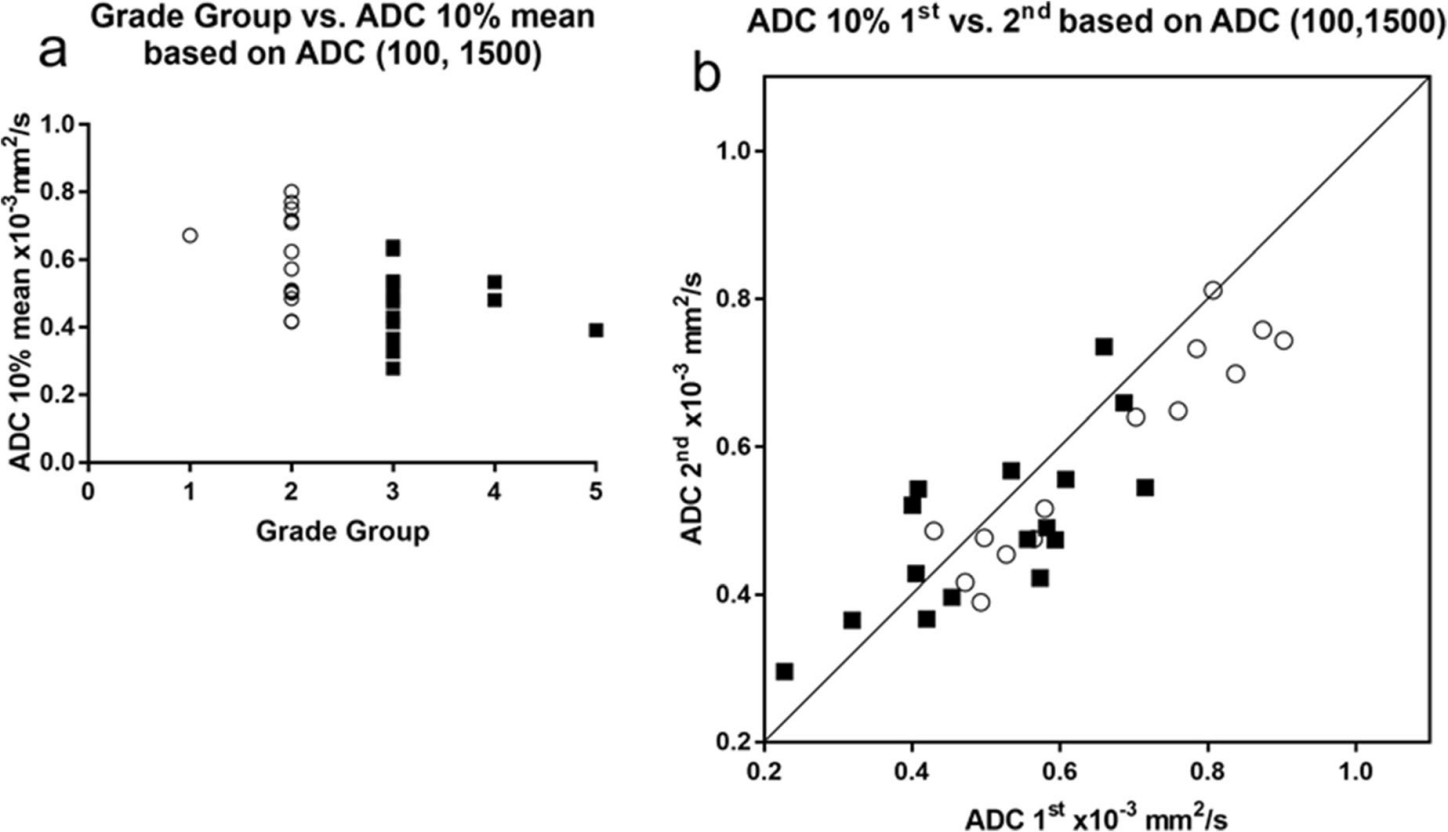
Peripheral zone cancer. **a** Correlation between grade group and mean of the first and second apparent diffusion coefficient (ADC) 10% based on ADC (100, 1,500). **b ***XY* plot of the first and second ADC 10% based on ADC (100, 1,500). Open circle, closed square, and line indicate grade group (GG) of 1 and 2, GG of 3, 4, and 5, and *Y* = *X* line, respectively

In PZ cancer, the following metrics showed significant correlation with GG at both examinations: ADC 10% and 25% based on ADC (0, 1,000); ADC 5%, 10%, and 25% based on ADC (0, 1,500); ADC 10%, 25%, and 50% based on ADC (100, 1,000); and ADC 5%, 10%, and 25% based on ADC (100, 1,500). Other metrics including higher-order texture features did not show significance. The results, including Spearman's ρ and its 95% confidence interval, the AUC of ROC for differentiation between GG of 1 and 2 *versus* GG of 3, 4, and 5, ICC and its 95% confidence interval, and Bland-Altman analysis (%) (bias, standard deviation of bias, 95% limit of agreement), are summarized in Table 3. Among them, as ADC 10%-based on ADC (0, 1,500) and ADC (100, 1,500) as well as ADC 25% based on ADC (100, 1,500) showed moderate correlation coefficient with GG (|ρ| > 0.4, *p* < 0.05), acceptable discrimination ability (AUC > 0.7) at both examinations, and good data repeatability (ICC > 0.8). The correlation between GG and ADC 10% mean of the first and second examinations based on ADC (100, 1,500), and their XY plot are shown in Fig. 4. To demonstrate the difference between the PZ and TZ, the correlation between GG and ADC 10% mean of the first and second examinations based on ADC (100, 1,500), and their XY plot in TZ cancer are also shown in Supplemental Fig. S3.

**Fig. 5:**
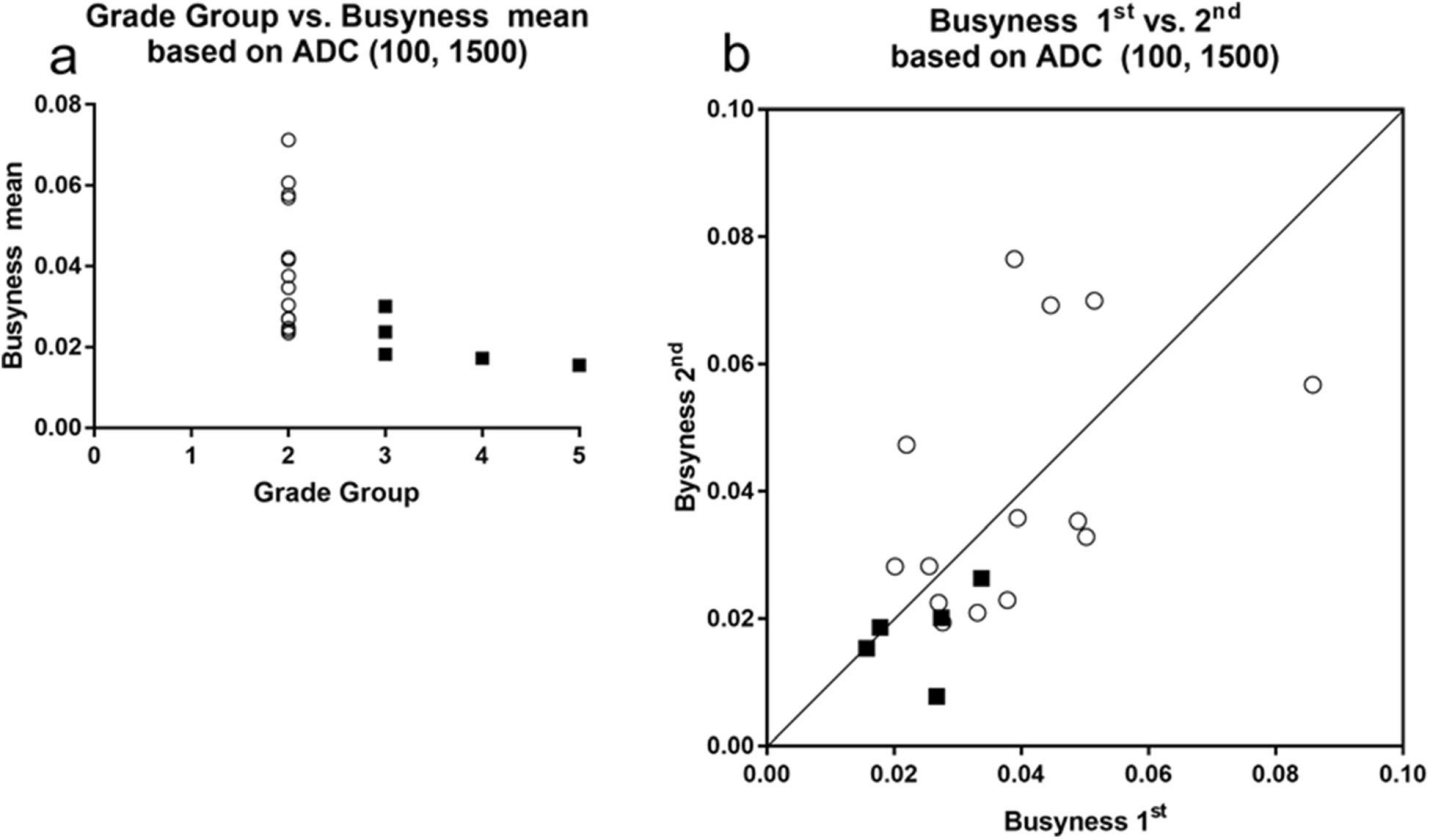
Transition zone cancer. **a** Correlation between grade group and mean of the first and second busyness of neighborhood gray-level difference matrix based on apparent diffusion coefficient (ADC) (100, 1,500). **b ***XY* plot of the first and second busyness of neighborhood gray-level difference matrix based on ADC (100, 1,500). Open circle, closed square, and line indicate grade group (GG) of 1 and 2, GG of 3, 4, and 5, and *Y* = *X* line, respectively

**Fig. 6:**
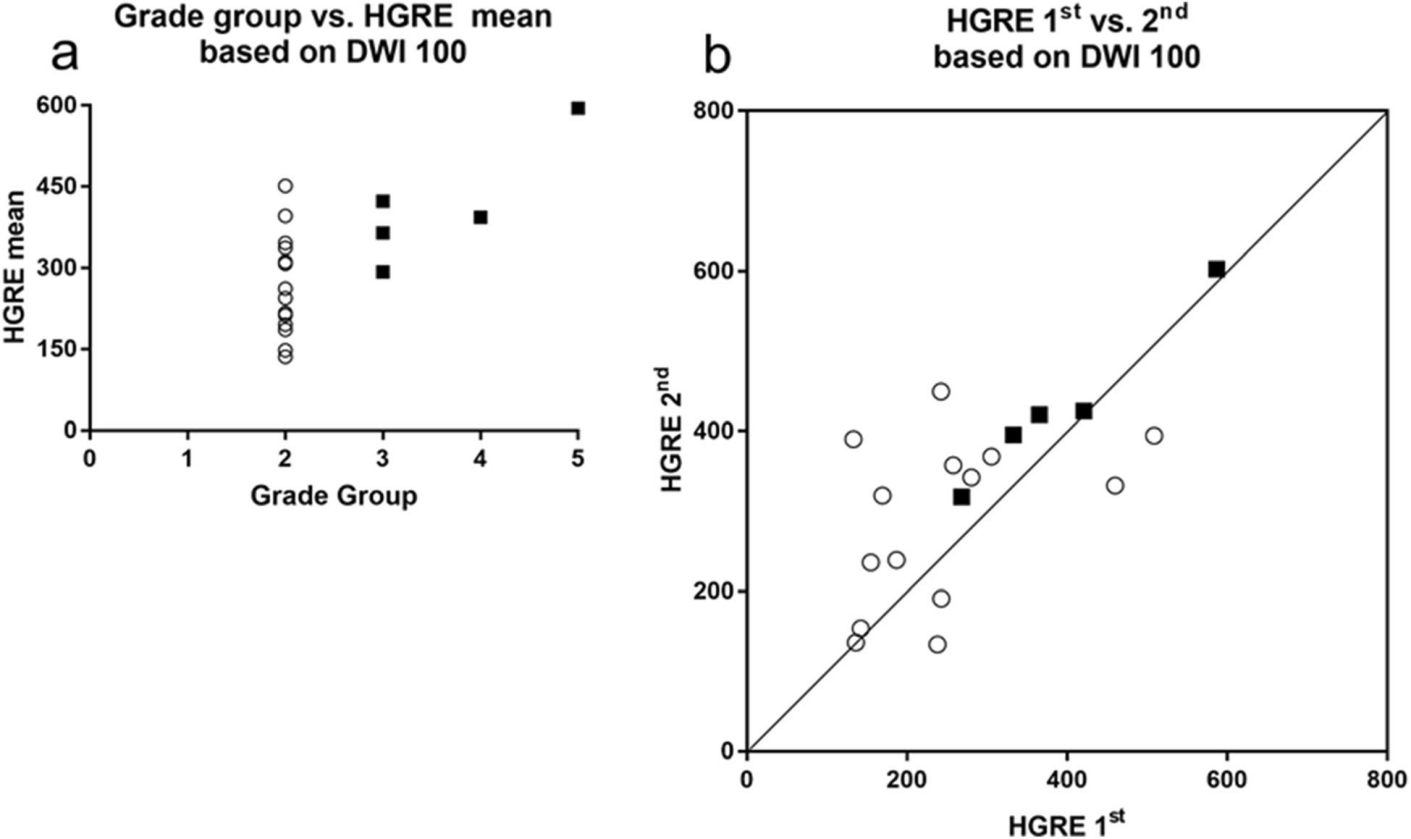
Transition zone cancer. **a** Correlation between grade group and mean of the first and second high gray-level run emphasis of gray-level run-length matrix based on diffusion-weighted (DWI) 100. **b** *XY* plot of the first and second high gray-level run emphasis of gray-level run-length matrix based on DWI 100. Open circle, closed square, and line indicate grade group (GG) of 1 and 2, GG of 3, 4, and 5, and Y = X line, respectively

For TZ cancer, SRHGE and busyness based on ADC (100, 1,500), and skewness, HGRE, SRHGE, LRHGE, HGZE, SZHGE, and busyness based on DWI 100, and skewness, HGRE, SRHGE, and HGZE based on DWI 0 showed significant correlations with GG. As opposed to PZ, the first-order statistical metrics did not show significance. The results, including Spearman's ρ and its 95% confidence interval, the ROC-AUC of for differentiating GG of 1 and 2 from GG of 3, 4, and 5, ICC and its 95% confidence interval, and Bland-Altman analysis (%) (bias, standard deviation of bias, 95% limit of agreement) are summarized in Table 4. Among them, busyness based on ADC (100, 1,500), and HGRE, SRHGE, and HGZE based on DWI 100 demonstrated moderate correlation coefficients with GG (|ρ| > 0.5, *p* < 0.05), excellent discrimination ability (AUC > 0.8) at both examinations, and moderate data repeatability (ICC > 0.5; skewness based on DWI 100 or 0 was excluded due to large standard deviation [> 300%] in Bland-Altman analysis [%]). The correlation between GG, and busyness mean of the first and second examinations based on ADC (100, 1,500) and HGRE mean of the first and second examinations based on DWI 100, and their XY plot are shown in Figs. 5 and 6, respectively.

## Discussion

To our knowledge, this is the first study showing a difference in DWI-related texture features that demonstrate not only significant correlations with GG and discrimination ability between GG of 1 and 2, *versus* GG of 3, 4, and 5, but also practical data repeatability between the PZ and TZ in prostate cancer.

In PZ cancer, ADC 10% based on ADC (0, 1,500) and (100, 1,500) as well as ADC 25% based on ADC (100, 1,500) satisfied moderate correlation and had acceptable discrimination and good repeatability. These results were in accordance with a systematic review reporting that ADC correlated moderately with GS (correlation coefficient of -0.48, 95% confidence interval of -0.54 to -0.42) [15]. However, Hectors SJ et al. [20] reported that SRE and LRE using bin 16 extracted from ADC map showed significance with GS. Several differences, such as analyzing the PZ and TZ together, calculating ADC with four *b*-values (0, 1,000, 1,600, and 2,000 s/mm^2^), and measuring texture feature using different methods, could explain the differences. Baek T et al. [21] reported that the entropy of GLCM from ADC map generated from *b*-values of 0 and 1,000 s/mm^2^ showed significance with GS. The differences in analyzing the PZ and TZ together and the distribution of lesion aggressiveness (16 out of total 65 lesions were GS of 6), including 19 biopsy-proven lesions, might explain the discrepancy. When analyzed by combining PZ and TZ cancers, the entropy of GLCM based on ADC (0, 1,000) did not show significance either in bin of 8, 16, or 32 setting (Supplemental Table S2).

In TZ cancer, busyness based on ADC (100, 1,500), and HGRE, SRHGE, and HGZE based on DWI 100 demonstrated moderate correlation coefficients, excellent discrimination, and moderate data repeatability. To evaluate the effect of bin number, texture features using bin 8 and 16 were also analyzed. Similar results were obtained (Supplemental Tables S3 and S4). In general, texture features for TZ cancer tend to show higher correlation and discrimination but lower data repeatability than those for PZ cancer.

Another important finding is that ADC histogram metrics such as 10%, which showed significance in PZ cancer, showed no significance in TZ cancer (Supplemental Fig. S3). This result was not inconsistent with the results of a systematic review, which reported that ADC correlated weakly (correlation coefficient of -0.22, 95% confidence interval of -0.47 to + 0.03) with GS in TZ cancer [15]. Furthermore, ADC 10% did not show significance and some features from DWI 100 and 0 demonstrating significance in TZ cancer may indirectly support that PI-RADS 2.1 puts emphasis on the findings of T2-weighted imaging for TZ cancer, because DWI with low *b*-value looks similar to fat-saturated T2-weighted imaging. However, it is unclear why DWI-related features showing significance with GG differ between PZ and TZ. One possible explanation might be that while the volume of the lumen and stroma is positively correlated with ADC, that of the epithelium is negatively correlated [22], and the degree of each composition differs between the PZ and TZ [23]. This may explain the results. However, the detailed mechanism underlying this is unknown.

Regarding which two *b*-value combination is appropriate for calculating ADC, ADC generated from DWI 100 and 1,500 would be relevant in terms of a correlation with GG (Tables 3 and 4). We cannot interpret these results with reasonable model and/or relevant hypothesis at this time but image quality improvement of DWI 1,500 due to performance advance of MRI-system would contribute to these results. In a study comparing diagnostic ability of prostate cancer based on DWI-related features, ADC value calculated from DWIs with *b*-values of 50 and 1,500 s/mm^2^ using a mono-exponential method reported to show the highest AUC among the IVIM, kurtosis, and IVIM-kurtosis methods [24], which is consistent with ours.

**Table 3:** Summary of the correlation between features and grade group as well as the repeatability in peripheral zone cancer

Base image/features	First/second	Grade group			Grade group 1, 2 *vs*. 3, 4, 5	ICC	95% CI	Bland-Altman plot (%)		
		Spearman's ρ	95% CI	*p*	AUC of ROC			Bias	SD of bias	95% LOA
ADC (0, 1,000)										
ADC 10%	First	-0.4027	-0.6725 to -0.03854	0.0273	0.732	0.773	0.580 to 0.885	-0.2016	21.15	-41.65 to 41.25
	Second	-0.4241	-0.6864 to -0.06427	0.0195	0.737					
ADC 25%	First	-0.3638	-0.6467 to 0.007035	0.0481	0.705	0.825	0.667 to 0.912	0.06553	15.54	-30.40 to 30.53
	Second	-0.411	-0.6779 to -0.04843	0.0241	0.728					
ADC (0, 1,500)										
ADC 5%	First	-0.3738	-0.6534 to -0.004499	0.0419	0.705	0.817	0.654 to 0.908	-8.692	19.58	-47.08 to 29.69
	Second	-0.4389	-0.6959 to -0.08239	0.0152	0.759					
ADC 10%	First	-0.418	-0.6824 to -0.05691	0.0215	0.737	0.829	0.674 to 0.915	-7.875	15.9	-39.04 to 23.29
	Second	-0.463	-0.7111 to -0.1123	0.01	0.763					
ADC 25%	First	-0.4035	-0.673 to -0.03941	0.027	0.714	0.865	0.738 to 0.933	-5.203	11.99	-28.71 to 18.31
	Second	-0.3736	-0.6532 to -0.004216	0.042	0.71					
ADC (100, 1,000)										
ADC 10%	First	-0.4083	-0.6761 to -0.04522	0.0251	0.732	0.75	0.542 to 0.872	-6.843	21.59	-49.16 to 35.47
	Second	-0.5043	-0.7368 to -0.1652	0.0045	0.786					
ADC 25%	First	-0.4103	-0.6774 to -0.04755	0.0243	0.723	0.786	0.601 to 0.892	-4.467	15.91	-35.66 to 26.73
	Second	-0.4555	-0.7064 to -0.1029	0.0114	0.754					
ADC 50%	First	-0.3794	-0.6571 to -0.01101	0.0387	0.692	0.82	0.658 to 0.910	-3.982	11.12	-25.77 to 17.81
	Second	-0.4013	-0.6715 to -0.0368	0.028	0.714					
ADC (100, 1,500)										
ADC 5%	First	-0.3903	-0.6643 to -0.02385	0.033	0.719	0.816	0.651 to 0.907	-4.456	18.31	-40.35 to 31.44
	Second	-0.4142	-0.6799 to -0.05222	0.0229	0.746					
ADC 10%	First	-0.4275	-0.6886 to -0.06841	0.0184	0.741	0.839	0.692 to 0.920	-6.85	12.82	-31.97 to 18.27
	Second	-0.4591	-0.7087 to -0.1074	0.0107	0.763					
ADC 25%	First	-0.4528	-0.7047 to -0.09954	0.012	0.746	0.843	0.699 to 0.922	- 5.629	10.96	-27.10 to 15.85
	Second	-0.4039	-0.6733 to -0.03999	0.0268	0.732					

*95% CI* 95% confidence interval, *95% LOA* 95% Limit of agreement, *AUC* Area under the curve, *ICC* Intraclass correlation coefficient, *ROC* Receiver operating characteristic, *SD* Standard deviation

**Table 4:** Summary of the correlation between features and grade group as well as the repeatability in transition zone cancer

Base image/features	First/second	Grade group			Grade group 1, 2 *vs*. 3, 4, 5	ICC	95% CI	Bland-Altman plot (%)		
		Spearman's ρ	95% CI	*p*	AUC of ROC			Bias	SD of bias	95% LOA
ADC (100, 1,500), bin 32										
SRHGE	First	0.488	0.02898 to 0.7771	0.034	0.814	0.427	-0.011 to 0.73	-21.4	27.77	-75.83 to 33.04
	Second	0.6912	0.3326 to 0.8752	0.001	0.943					
busyness	First	-0.5039	-0.7853 to -0.05002	0.0278	0.814	0.576	0.188 to 0.811	21.1	77.36	-130.5 to 172.7
	Second	-0.6742	-0.8675 to -0.3039	0.0015	0.929					
DWI 100, bin 32										
skewness	First	-0.5232	-0.7951 to -0.07612	0.0215	0.829	0.629	0.266 to 0.837	-28.03	310.3	-636.2 to 580.1
	Second	-0.4699	-0.7676 to -0.005422	0.0424	0.786					
HGRE	First	0.5663	0.1368 to 0.8166	0.0115	0.857	0.658	0.312 to 0.852	-14.68	35.99	-85.22 to 55.85
	Second	0.5516	0.1157 to 0.8093	0.0144	0.843					
SRHGE	First	0.5663	0.1368 to 0.8166	0.0115	0.857	0.619	0.251 to 0.833	-15.33	35.93	-85.76 to 55.1
	Second	0.5902	0.1717 to 0.8282	0.0078	0.871					
LRHGE	First	0.4892	0.03047 to 0.7777	0.0335	0.8	0.802	0.563 to 0.918	-12.04	37.52	-85.58 to 61.5
	Second	0.4744	0.01126 to 0.77	0.0401	0.786					
HGZE	First	0.5277	0.08236 to 0.7974	0.0202	0.829	0.558	0.161 to 0.802	-13.03	32.89	-77.5 to 51.44
	Second	0.5277	0.08236 to 0.7974	0.0202	0.829					
SZHGE	First	0.5516	0.1157 to 0.8093	0.0144	0.843	0.373	-0.075 to 0.699	-14.48	33.09	-79.34 to 50.38
	Second	0.6072	0.1974 to 0.8364	0.0058	0.886					
busyness	First	-0.6151	-0.8401 to -0.2095	0.0051	0.900	0.548	0.148 to 0.797	9.866	44.39	-77.14 to 96.87
	Second	-0.4642	-0.7646 to 0.001836	0.0453	0.771					
DWI 0, bin 32										
skewness	First	-0.5743	-0.8205 to -0.1483	0.0101	0.871	0.575	0.185 to 0.81	-55.02	303.2	-649.3 to 539.2
	Second	-0.4982	-0.7824 to -0.04246	0.0299	0.800					
HGRE	First	0.5947	0.1785 to 0.8304	0.0072	0.871	0.579	0.192 to 0.813	-14.98	34.32	-82.24 to 52.28
	Second	0.4721	0.008339 to 0.7688	0.0412	0.786					
SRHGE	First	0.614	0.2078 to 0.8396	0.0052	0.886	0.537	0.132 to 0.791	-15.22	34.93	-83.67 to 53.24
	Second	0.4892	0.03047 to 0.7777	0.0335	0.8					
HGZE	First	0.5947	0.1785 to 0.8304	0.0072	0.871	0.524	0.115 to 0.784	-10.79	32.12	-73.75 to 52.18
	Second	0.4937	0.03645 to 0.78	0.0317	0.8					

*95% CI* 95% confidence interval, *95% LOA* 95% Limit of agreement, *AUC* Area under the curve, *HGRE* High gray-level run emphasis, *HGZE* High gray-level zone emphasis*, ICC* Intraclass correlation coefficient, *LRHGE* Long-run high gray-level emphasis*, ROC* Receiver operating characteristic, *SD* Standard deviation, *SRHGE* Short-run high gray-level emphasis*, SZHGE* Short-zone high gray-level emphasis

Another focus of the present study is data repeatability. DWI-related features with significance for PZ cancer demonstrated good repeatability, but those for TZ cancer remained moderate. However, moderate repeatability may be acceptable in clinical practice. In a previous study, the κ value for the reproducibility of the PI-RADS 2 score in TZ was 0.525 [25]. In another study, ICCs of lesion size in the TZ were 0.80 and 0.58 for intra-reader and inter-reader analyses, respectively [26].

Texture features themselves have high potential with respect to correlation with lesion aggressiveness and clinical outcome. However, those have a tendency prone to be affected by a mild difference of the imaging data including artifacts. Therefore, reliability studies not only for observers but also for imaging data themselves should be verified sufficiently before being applied to clinical practice.

This study has some limitations. First, we analyzed patients who underwent radical prostatectomy because of the clear correlation between histology and mpMRI, but this concept would have reduced the number of the cases and lesions included in the study. Second, texture features of T2-weighted imaging were not evaluated because matrix size was different from DWI and the voxel number in the ROI differed greatly. Third, texture features were extracted from two-dimensional ROI because the lesion size was not large enough to extract features from three-dimensional ROI. Fourth, ROI assignment was performed by consensus between two observers, not carried out independently. We consider consensus reading would be acceptable because one of the main purposes of the present study was to assess reliability of the imaging data themselves. Finally, in both PZ and TZ cancer, the number of lesions was not sufficiently large; therefore, further analyses by combining features through logistic regression and/or discriminant analyses were not performed.

In conclusion, some DWI-related features showed significant correlation with GG and clinically acceptable data repeatability in histologically confirmed prostate cancer, and they differed between the PZ and TZ. The texture features for TZ cancer tended to show higher correlation with GG and higher discrimination ability between GG of 1 and 2 *versus* GG of 3, 4, and 5, but lower data repeatability than those for PZ cancer. Regarding a correlation with GG, DWI 100 and 1,500 s/mm^2^, and ADC generated from these two images would be relevant.

## Supplementary Information

Additional file 1: Supplemental Table 1 Acquisition parameters for diffusion-weighted imaging

## Data Availability

The datasets used and/or analyzed during the current study are available from the corresponding author on reasonable request.

## Abbreviations

AUC: Area under the curve
GG: Grade group
GLCM: Gray-level co-occurrence matrix
GLZSM: Gray-level zone-size matrix
GS: Gleason score
HGRE: High gray-level run emphasis
HGZE: High gray-level zone emphasis
ICC: Intraclass correlation coefficient
LRE: Long-run emphasis
LRHGE: Long-run high gray-level emphasis
mpMRI: Multiparametric MRI
MRI: Magnetic resonance imaging
NGLDM: Neighborhood gray-level difference matrix
PI-RADS: Prostate Imaging–Reporting and Data System
PZ: Peripheral zone
ROC: Receiver operating characteristic
ROI: Region of interest
SRE: Short-run emphasis
SRHGE: Short-run high gray-level emphasis
SZE: Short-zone emphasis
SZHGE: Short-zone high gray-level emphasis
TZ: Transition zone
