# Correction: DWI-related texture analysis for prostate cancer: differences in correlation with histological aggressiveness and data repeatability between peripheral and transition zones

**DOI:** 10.1186/s41747-022-00268-y

**Published:** 2022-03-23

**Authors:** Chie Tsuruta, Kenji Hirata, Kohsuke Kudo, Naoya Masumori, Masamitsu Hatakenaka

**Affiliations:** 1 Department of Diagnostic Radiology, School of Medicine, Sapporo Medical University, South 1, West 17, Chuo-ku, Sapporo, 060-8556 Japan; 2 Department of Diagnostic Imaging, Graduate School of Medicine, Hokkaido University, Sapporo, Japan; 3 Global Center for Biomedical Science and Engineering, Faculty of Medicine, Hokkaido University, Sapporo, Japan; 4 Department of Urology, School of Medicine, Sapporo Medical University, Sapporo, Japan

**Correction: Eur Radiol Exp 6, 1-12 (2022)**

**https://doi.org/10.1186/s41747-021-00252-y**

The original article contained minor errors affecting citations and references which have since been corrected.

