# Supplementary material for: DWI-related texture analysis for prostate cancer: differences in correlation with histological aggressiveness and data repeatability between peripheral and transition zones
Source: Eur Radiol Exp. 2022 Jan 12;6:1. doi: 10.1186/s41747-021-00252-y (PMC8752657; doi:10.1186/s41747-021-00252-y)

**ELECTRONIC SUPPLEMENTARY MATERIAL**

**Supplemental Table 1**

**Acquisition parameters for diffusion-weighted imaging**

| Repetition time (ms) | 3200-4200 |
| --- | --- |
| Echo time (ms) | 73.98-74.29 |
| Field of view (mm) | 200 × 200 |
| Matrix, acquisition / reconstruction | 80 × 79 / 256 × 256 |
| Slice thickness/Gap (mm) | 3 / 0 |
| b-values (s/mm^2^)/number of averages | 0 / 3, 100 / 3, 1000 / 9, 1500 / 12 |
| Acquisition time | 4 min 12 s~5 min 22 s |

| **Supplemental Table 2 Correlation between entropy of GLCM and grade group** | | | | |
| --- | --- | --- | --- | --- |
|  |  |  |  |  |
| **Base image/ Features** | **First / Second** | **Grade group** | | |
| ADC (0, 1000) |  | Spearman r | 95% CI | p |
| bin=8 | First | 0.111 | -0.184 to 0.388 | 0.447 |
|  | Second | -0.039 | -0.325 to 0.253 | 0.789 |
| bin=16 | First | 0.022 | -0.269 to 0.309 | 0.394 |
|  | Second | -0.201 | -0.463 to 0.094 | 0.166 |
| bin=32 | First | -0.032 | -0.318 to 0.259 | 0.827 |
|  | Second | -0.135 | -0.408 to 0.161 | 0.356 |

*95% CI* 95% confidence interval, *GLCM* gray-level co-occurrence matrix

| **Supplemental Table 3 Correlation between features using bin of 8 and grade group** | | | | |
| --- | --- | --- | --- | --- |
|  |  |  |  |  |
| **Base image/ Features** | **First / Second** | **Grade group** | | |
| ADC (100, 1500) |  | Spearman r | 95% CI | p |
| HGRE | First | 0.4687 | 0.00397 to 0.767 | 0.0429 |
|  | Second | 0.6912 | 0.3326 to 0.8752 | 0.001 |
| SRHGE | First | 0.4789 | 0.01714 to 0.7723 | 0.038 |
|  | Second | 0.5902 | 0.1717 to 0.8282 | 0.0078 |
| busyness | First | -0.5016 | -0.7841 to -0.04699 | 0.0286 |
|  | Second | -0.7127 | -0.8847 to -0.3699 | 0.0006 |
| DWI 100, |  |  |  |  |
| HGRE | First | 0.5663 | 0.1368 to 0.8166 | 0.0115 |
|  | Second | 0.5323 | 0.08862 to 0.7997 | 0.019 |
| SRHGE | First | 0.6049 | 0.1939 to 0.8353 | 0.0061 |
|  | Second | 0.656 | 0.2741 to 0.8592 | 0.0023 |
| LRLGE | First | -0.5119 | -0.7894 to -0.06069 | 0.0251 |
|  | Second | -0.5539 | -0.8105 to -0.1189 | 0.0139 |
| DWI 0 |  |  |  |  |
| HGRE | First | 0.537 | 0.11 to 0.7966 | 0.0146 |
|  | Second | 0.4597 | -0.007607 to 0.7622 | 0.0477 |
| SRHGE | First | 0.6123 | 0.2195 to 0.8343 | 0.0041 |
|  | Second | 0.4755 | 0.01273 to 0.7706 | 0.0396 |

*95% CI* 95% confidence interval, *HGRE* high gray-level run emphasis, *LRLGE* long-run low gray-level emphasis, *SRHGE* short-run high gray-level emphasis

| **Supplemental Table 4 Correlation between features using bin of 16 and grade group** | | | | |
| --- | --- | --- | --- | --- |
|  |  |  |  |  |
| **Base image/ Features** | **First / Second** | **Grade group** | | |
| ADC (100, 1000) |  | Spearman r | 95% CI | p |
| ZLNU | First | 0.4631 | -0.003281 to 0.764 | 0.0459 |
|  | Second * | -0.5516 | -0.8093 to -0.1157 | 0.0144 |
| ADC (100, 1500) |  |  |  |  |
| busyness | First | -0.4971 | -0.7818 to -0.04095 | 0.0304 |
|  | Second | -0.7638 | -0.9069 to -0.4628 | 0.0001 |
| DWI 100 |  |  |  |  |
| skewness | First | -0.4846 | -0.7753 to -0.02452 | 0.0355 |
|  | Second | -0.4892 | -0.7777 to -0.03047 | 0.0335 |
| HGRE | First | 0.5663 | 0.1368 to 0.8166 | 0.0115 |
|  | Second | 0.5516 | 0.1157 to 0.8093 | 0.0144 |
| SRHGE | First | 0.5709 | 0.1434 to 0.8188 | 0.0107 |
|  | Second | 0.6333 | 0.2378 to 0.8487 | 0.0036 |
| HGZE | First | 0.4892 | 0.03047 to 0.7777 | 0.0335 |
|  | Second | 0.5448 | 0.106 to 0.806 | 0.0159 |
| busyness | First | -0.5766 | -0.8216 to -0.1516 | 0.0098 |
|  | Second | -0.4812 | -0.7735 to -0.02009 | 0.037 |
| DWI 0 |  |  |  |  |
| skewness | First | -0.5743 | -0.8205 to -0.1483 | 0.0101 |
|  | Second | -0.4982 | -0.7824 to -0.04246 | 0.0299 |
| HGRE | First | 0.6095 | 0.2008 to 0.8374 | 0.0056 |
|  | Second | 0.4721 | 0.008339 to 0.7688 | 0.0412 |
| SRHGE | First | 0.6095 | 0.2008 to 0.8374 | 0.0056 |
|  | Second | 0.5085 | 0.05611 to 0.7876 | 0.0262 |
| *Spearman r values are in the opposite direction. | | |  |  |

*95% CI* 95% confidence interval, *HGRE* high gray-level run emphasis, *HGZE* high gray-level zone emphasis, *SRHGE* short-run high gray-level emphasis, *ZLNU* zone length non-uniformity

**Supplemental Figure 1**

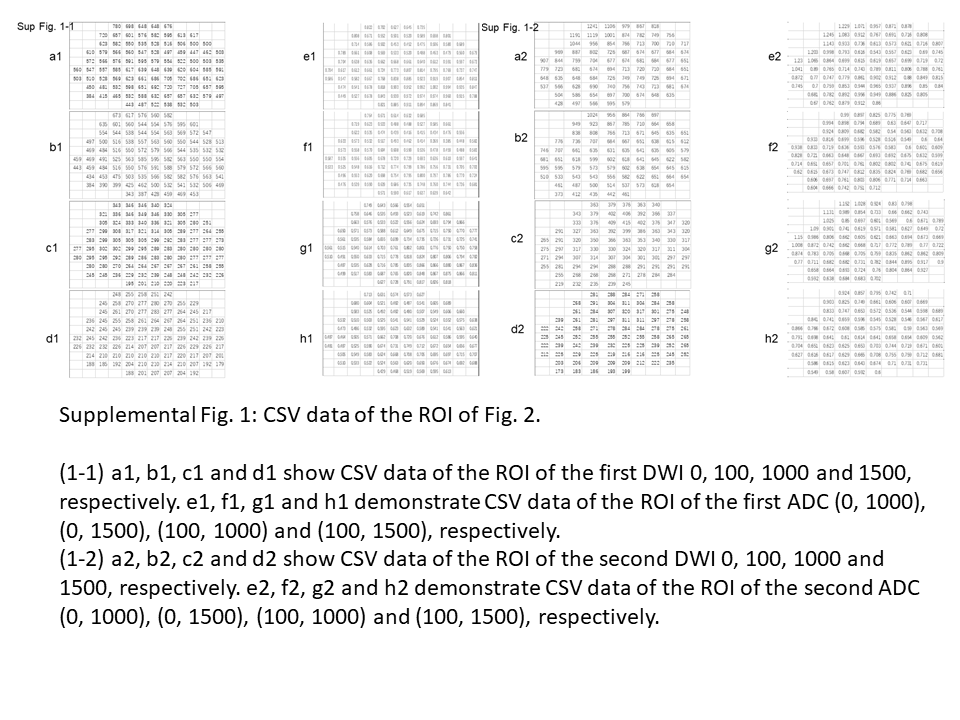


**Supplemental Figure 2**


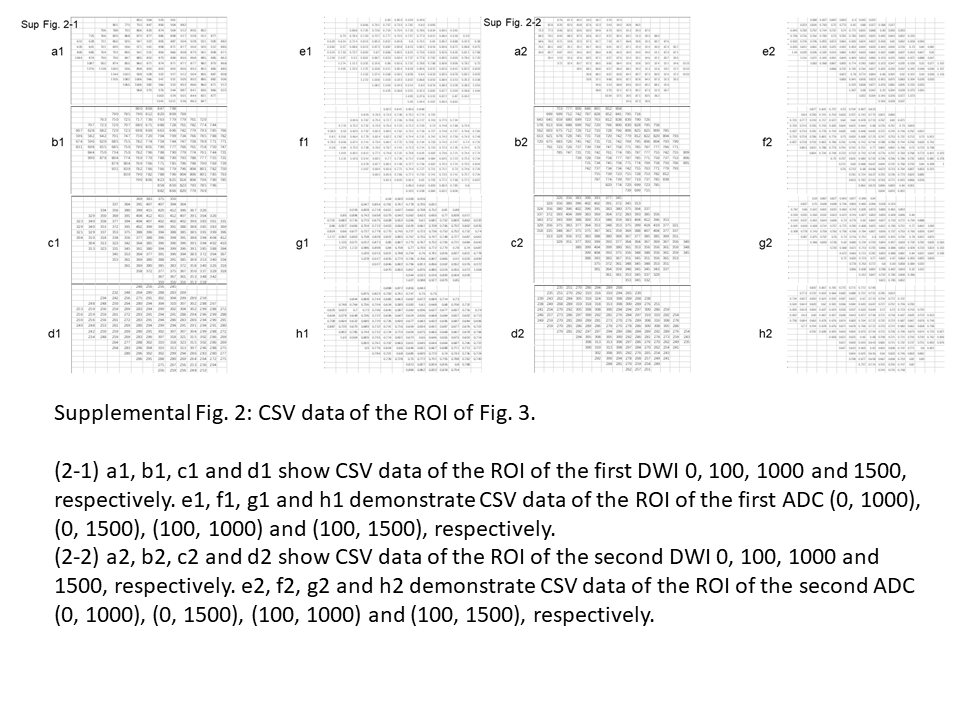


**Supplemental Figure 3**

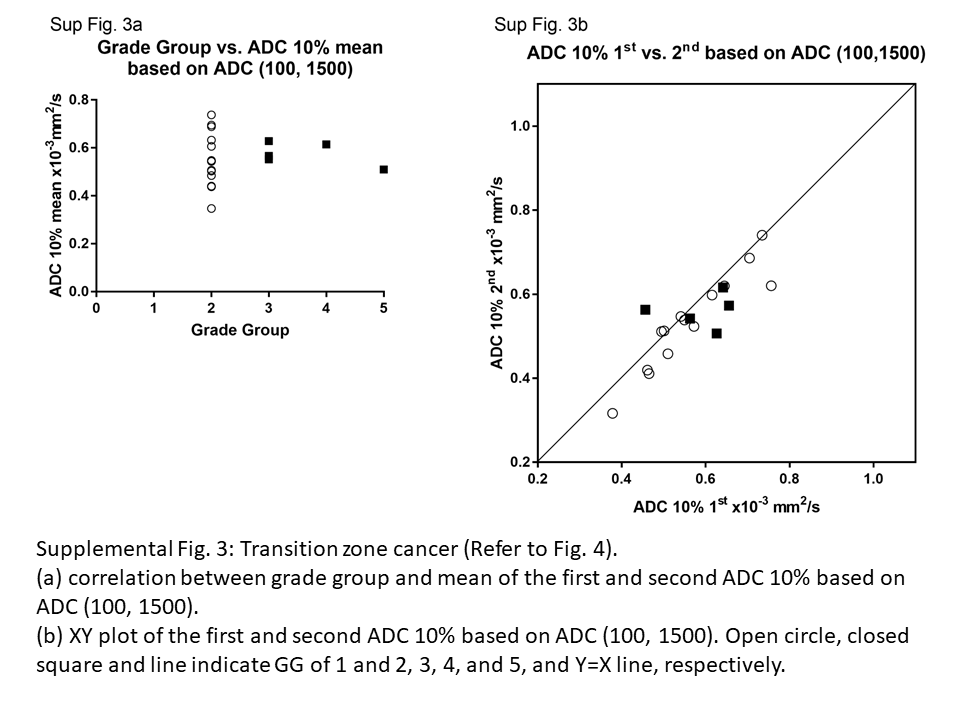

Supplement: Supplementary file 1 — Supplemental Table 1 Acquisition parameters for diffusion-weighted imaging [file 41747_2021_252_MOESM1_ESM.docx]
